# Editorial: Secondary vs. Idiopathic Autism

**DOI:** 10.3389/fpsyt.2020.00297

**Published:** 2020-04-14

**Authors:** Manuel F. Casanova, Emily L. Casanova, Richard Eugene Frye, Carolina Baeza-Velasco, Janine M. LaSalle, Randi Jensen Hagerman, Stephen W. Scherer, Marvin R. Natowicz

**Affiliations:** ^1^School of Medicine Greenville, University of South Carolina, Greenville, SC, United States; ^2^Phoenix Children’s Hospital, Phoenix, AZ, United States; ^3^Université Paris Descartes, Île-de-France, France; ^4^University of California, Davis, Davis, CA, United States; ^5^Department of Pediatrics, UC Davis School of Medicine, Sacramento, CA, United States; ^6^University of Toronto, Toronto, ON, Canada; ^7^Cleveland Clinic, Cleveland, OH, Canada

**Keywords:** autism spectrum disorder, idiopathic autism, secondary autism, Fragile X syndrome, anxiety, seizures

This Research Topic consists of four articles, contributed by 30 authors, each focusing on various aspects of secondary autism spectrum disorder (ASD). The articles in this Research Topic discuss some of the controversies and progress made in how to assess a child with secondary ASD. They discuss the utility of using genetic and metabolic tests, as well as combinations of the same. They also grapple with the relationship of intellectual disability (ID) and other symptoms in patients with secondary ASD.

The diagnostic boundaries of the behavioral phenotype that define ASD are fairly broad due to the large variability that is observed in symptom types, onset, and severity. This variability serves as an index of etiological heterogeneity for a group of complex conditions where multiple genetic/environmental hits provide an additive burden to symptom expression. Within this spectrum of disorders our Research Topic examines a subpopulation of patients where the ASD phenotype occurs and a specific cause can be identified (1). These cases are often referred to as “secondary” ASD as a way of distinguishing them from “idiopathic” ASD. While there is some variation of the estimates, about 85% of individuals with ASD are termed as having idiopathic ASD with about 15% diagnosed with secondary ASD (2). Cases of secondary ASD display significant etiological diversity and, sometimes, more severe behavioral problems than individuals having idiopathic ASD (3, 4). Well documented causes of secondary ASD include conditions such as tuberous sclerosis, down syndrome, Fragile X syndrome (FXS), and some congenital infections (e.g., cytomegalovirus) (5–7).

Differentiating between idiopathic and secondary ASD is essential when drawing conclusions regarding pharmacological and behavioral treatments among published studies (8). Clinical study design benefits from the selection of homogeneous populations, those without prognostically distinct subgroups, as it makes it easier to assess treatment efficacy (*vide infra*, (9)]. Indeed, a thorough understanding of the different clinical presentations, natural history and even life expectancies of persons with ASD should go beyond the influence of traditional variables and use relevant classifiers of biomedical and sociocultural differences that may have an impact on the health and wellbeing of the studied subject population.

Genomic scanning is beginning to elucidate the underlying genetic architecture that confers ASD susceptibility. Chromosomal and penetrant gene abnormalities are common, contributing to about 5%–15% of ASD cases in some estimates (10). By way of contrast, *de novo* chromosomal copy number variants, important contributors to lower IQ, and diminished motor skills (11), are seen in approximately 5% of persons diagnosed with idiopathic ASD (Glinton and Elsea). Many additional cases have DNA sequence variants in genes whose pathology and function provide an uncertain contribution to ASD. These genetic modifications are described as variants with unknown significance (VUS) (12).

For disorders associated with secondary ASD, there is a substantial increase in the prevalence rate of ASD as compared to the general population. The underlying reason for this association is complex and may include a disruption of brain function caused by the superimposed pathology, the possible linkage between ASD risk genes and those of the secondary condition (e.g., ID), and, for some, the commonality in the expression of seizures and their effects on brain development (4, Thurm et al.). Establishing a diagnosis of ASD in this patient population is complicated by the fact that traits of ASD often exhibit a phenotypic and genetic overlap with symptoms of other disorders. It is therefore unsurprising that affected individuals often live with challenges that may confound behavioral observations (Thurm et al.). Indeed, these patients may commonly present with ID, seizures and/or anxiety in addition to comorbid ASD symptoms (11). Identification in these cases often depends on clinical suspicion, especially in severe phenotypes, and both metabolic and genome-wide screening (Glinton and Elsea).

We suggest a concerted effort at developing integrated surveys of clinical, metabolic, and genomic data as a background tool for patient stratification. By establishing a proper diagnosis these tools would aid in understanding underlying pathogenetic mechanisms, in customizing medical/treatment decisions, in offering explanations towards some of the patients’ behaviors and, possibly, in providing knowledge helpful in guiding parents and professionals regarding needed school and community services. This integrated approach complements, when necessary, the recommended general developmental screening proposed by the American Academy of Pediatrics (12), keeping in mind that early intervention can and does influence outcomes (13).

Potter et al. reported a clinical trial of sertraline, a selective serotonin reuptake inhibitor, in nonsyndromic ASD. A previous controlled trial of low dose sertraline in children 2 to 6 years old with FXS had demonstrated significant benefit of sertraline compared to placebo in developmental measures on the Mullen Scales of Early Development, especially in the 60% who has ASD in addition to FXS (14). However, Potter et al. performed a randomized, double-blind, placebo-controlled study (n=32 sertraline, n=26 controls) in children with ASD without FXS aged 24-72 months that showed no benefit in primary or secondary outcome measures of language development.

In ASD, the heterogeneity observed along a spectrum of clinical presentations and natural histories is a sobering reminder of the difficulty ingrained in making comparisons among different patient populations. It is not surprising that results of studies using populations of syndromic [FXS, (14)] and idiopathic ASD (Potter et al.) may not reproduce each other. In these cases, large and well characterized study populations are needed in order to perform secondary analyses that could help elucidate differences in severity among the studied populations and better capture individual differences within and between subgroups of autistic individuals. This study concurs with the majority of clinical trials on selective serotonin reuptake inhibitors which show very mixed results and overall suggest that these medications may not be appropriate for children with ASD (15).

Glinton and Elsea offer a comprehensive review of metabolomics and the use of targeted/untargeted assays and their possible role in the early screening and diagnosis of secondary ASD caused by inborn errors of metabolism. The review also introduces the reader to the emerging field of nonspecific metabolic disturbances and how they can lead to clues on mechanisms and treatment. Considering the large variety of metabolic disturbances identified in ASD, the authors question the extent to which any biomarker would accurately assess risk or guide individual treatment. The answer to the question may lie in the use of untargeted assays in order to broadly survey the multiple aspects of the metabolism of each individual patient. The use of biomarkers as metabolic measurements to improve ASD diagnosis and identification of underlying causes is a promising research tool in the hands of physicians but requires specialized skills for proper interpretation (16).

Thurm et al. consider the impact of ID on the diagnosis of ASD. The authors provide a review of the conceptual evolution of autism diagnosis and the different tools used for this purpose. They propose the use of operationalized criteria for making the clinical distinction between ID with and without ASD and provide specific recommendation for testing depending on the degree of ID. The authors consider that the presence of ID in ASD is one of the strongest indicators that an associated condition may be present.

Lovato et al. clarifies the relevance of genetic variants of purported unknown significance (VUS). Some of these variants are genes associated with ASD, but the altered region lacks functional data linking it to pathogenicity. Other VUS are located in genes having few associations with ASD, but may seem relevant to the clinical phenotype. This review summarizes a strategy for obtaining information about VUS by focusing on both clinical phenotyping and a genetic curation process. It is hoped that advanced understanding of these genetic variants may help clarify risk and guide individual treatment options.

## Author Contributions

All authors listed have made a substantial, direct, and intellectual contribution to the work, and approved it for publication.

## Conflict of Interest

The authors declare that the research was conducted in the absence of any commercial or financial relationships that could be construed as a potential conflict of interest.
